# Extracellular Vesicles-Based Biomarkers Represent a Promising Liquid Biopsy in Endometrial Cancer

**DOI:** 10.3390/cancers11122000

**Published:** 2019-12-12

**Authors:** Carolina Herrero, Alexandre de la Fuente, Carlos Casas-Arozamena, Victor Sebastian, Martin Prieto, Manuel Arruebo, Alicia Abalo, Eva Colás, Gema Moreno-Bueno, Antonio Gil-Moreno, Ana Vilar, Juan Cueva, Miguel Abal, Laura Muinelo-Romay

**Affiliations:** 1 Translational Medical Oncology Group (Oncomet), Health Research Institute of Santiago de Compostela (IDIS), University Hospital of Santiago de Compostela (SERGAS), Trav. Choupana s/n, 15706 Santiago de Compostela, Spain; carolina.herrero@rai.usc.es (C.H.); carlos.casas_95@hotmail.es (C.C.-A.); Alicia.Abalo.Pineiro@sergas.es (A.A.); ana.vilar@telefonica.net (A.V.); jfcueva@gmail.com (J.C.); miguel.abal.posada@sergas.es (M.A.); 2 Nasasbiotech, S.L., Canton Grande 3, 15003 A Coruña, Spain; afuegon79@gmail.com; 3 Department of Chemical Engineering, Aragon Institute of Nanoscience (INA), University of Zaragoza, 50018 Zaragoza, Spain; victorse@unizar.es (V.S.); mprieto@unizar.es (M.P.); arruebom@unizar.es (M.A.); 4 Networking Research Center on Bioengineering, Biomaterials and Nanomedicine (CIBER-BBN), Monforte de Lemos 3–5, 28029 Madrid, Spain; 5 Biomedical Research Group in Gynecology, Vall Hebron Research Institute (VHIR), Universitat Autonoma de Barcelona, 119–129 Pg. Vall d’Hebron, 08035 Barcelona, Spain; eva.colas@vhir.org (E.C.); antonioimma@yahoo.com (A.G.-M.); 6 Centro de Investigación Biomédica en Red de Cáncer (CIBERONC), Monforte de Lemos 3–5, 28029 Madrid, Spain; gmoreno@iib.uam.es; 7 Department of Biochemistry, Universidad Autonoma de Madrid (UAM), Instituto de Investigaciones Biomedicas “Alberto Sols” (CSIC-UAM), IdiPaz, Pedro Rico, 6, 28029 Madrid, Spain; 8 Fundacion MD Anderson Internacional, C/Gómez Hemans 2, 28033 Madrid, Spain

**Keywords:** EVs, ExoGAG, endometrial cancer, ANXA2

## Abstract

Tumor-derived extracellular vesicles (EVs) are secreted in large amounts into biological fluids of cancer patients. The analysis of EVs cargoes has been associated with patient´s outcome and response to therapy. However, current technologies for EVs isolation are tedious and low cost-efficient for routine clinical implementation. To explore the clinical value of circulating EVs analysis we attempted a proof-of-concept in endometrial cancer (EC) with ExoGAG, an easy to use and highly efficient new technology to enrich EVs. Technical performance was first evaluated using EVs secreted by Hec1A cells. Then, the clinical value of this strategy was questioned by analyzing the levels of two well-known tissue biomarkers in EC, L1 cell adhesion molecule (L1CAM) and Annexin A2 (ANXA2), in EVs purified from plasma in a cohort of 41 EC patients and 20 healthy controls. The results demonstrated the specific content of ANXA2 in the purified EVs fraction, with an accurate sensitivity and specificity for EC diagnosis. Importantly, high ANXA2 levels in circulating EVs were associated with high risk of recurrence and non-endometrioid histology suggesting a potential value as a prognostic biomarker in EC. These results also confirmed ExoGAG technology as a robust technique for the clinical implementation of circulating EVs analyses.

## 1. Introduction

Endometrial cancer (EC) is the most common neoplasm of the female genital tract in the developed world, and the incidence has risen over the past years. Despite the progress in early detection and treatment, a significant number of cases of advanced ECs are still diagnosed. Consequently, mortality from this cancer has not improved in recent decades and is primarily driven by high-grade carcinomas that are more likely to present at an advanced stage and, ultimately, are more likely to recur. The prognosis for recurrent EC is poor, especially for the 50% of these advanced ECs that present extra-pelvic disease recurrence. To this regard, effort has been mainly dedicated to reach a consensus on EC risk classification to promote consistency for future clinical trials design, including their molecular characterization and its integration into clinicopathological profiling to develop prognostic and predictive biomarkers [1,2]. Various immunohistochemical markers have been studied for the prediction of recurrences, like loss of the estrogen receptor (ER) and progesterone receptor (PR). In particular, the presence of the L1 cell adhesion molecule (L1CAM) has been described as a strong independent predictor for distant recurrence and overall survival in EC, as well as a marker to select patients who could benefit from more extensive diagnostic and/or therapeutic procedures [3,4,5]. Likewise, annexin-A2 has been reported as a predictor of recurrent disease in EC and as an independent risk factor for poor prognosis [6,7,8]. Nevertheless, these biomarkers have not been implemented into clinical practice, in part due to limited sensitivity and/or specificity, absence of robust clinical validation and compromised performance in terms of cost-effectiveness.

In parallel to the discovery and the technical and clinical validation of soluble protein biomarkers, the concept of liquid biopsy refers to the analysis of tumoral material shed from primary tumors and their metastatic sites into peripheral blood like circulating tumor cells (CTCs), circulating tumor DNA (ctDNA), circulating miRNAs, and tumor-derived extracellular vesicles (EVs). Major advantages of liquid biopsies analysis include real-time information about the disease by minimal invasive procedures that can be performed regularly [9]. In particular, small extracellular vesicles (sEVs) secreted by tumor cells are revolutionizing the field of liquid biopsy, as safe carriers of nucleic acids, proteins, and lipids reflective of the disease state. As responsible for myriad roles in physiology and disease, and the main mediators of cell-to-cell communication between the tumor and stromal cells in both local and distant microenvironments, this heterogeneous group of small lipid-enclosed structures might be a relevant source for diagnostic and therapeutic tools [10]. The biological relevance of tumor EVs in disease progression and metastatic potential has been reported [11], with an increased secretion associated with high-grade disease [12]. Limited work has been reported in the field of EVs in EC [13], in particular on their phenotypic characterization and involvement in the metastatic fate of CTCs [14].

Despite its potential, widespread implementation of EV-based biomarkers in clinical practice has not been yet achieved. This is mainly due to the limited performance of current methodologies for the separation and enrichment of EVs for clinical and technical validation with sufficient specificity and sensitivity for routine clinical implementation [15]. Here, we describe a proof-of-concept to evaluate the clinical applicability of EV-based biomarkers for EC management with ExoGAG technology. EVs are decorated on their surface with glycoproteins associated with EV biogenesis and different biological functions [16]. ExoGAG technology is based on the ability of glycosaminoglycans (GAGs), that are negatively charged linear polysaccharides composed of repeating disaccharides with variable sulfation levels, to neutralize the positive charge of cationic agents leading to their aggregation in a complex that can be further purified by centrifugation [17]. We first assessed the ExoGAG technical performance by studying EVs purified from conditioned culture media, resulting in a robust and reproducible methodology with a simple and easy to handle protocol. Moreover, we further validated the ExoGAG clinical performance in plasma samples from EC patients by analyzing L1CAM and ANXA2, known biomarkers for this tumor, in the isolated EVs, resulting in an improved diagnostic and prognostic value.

## 2. Results

### 2.1. Purification of EVs from Conditioned Media from Endometrial Cancer Cells

**Figure 1:**
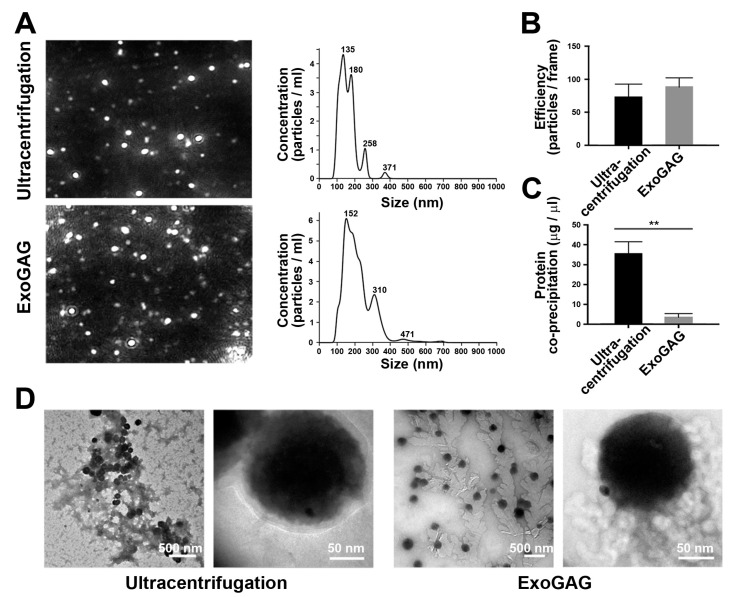
Characterization of Hec1A-derived extracellular vesicles (EVs) obtained by ultracentrifugation and ExoGAG. (**A**) Representative nanoparticle tracking analysis (NTA) Nanosight NS300 image of isolated EVs showing particle size (nm) and concentration (particles/mL)**.** A similar particle profile was obtained in both methodologies (*n* = 4). (**B**) Efficiency of purified EVs, expressed as the number of EVs per frame, was similar in ExoGAG and ultracentrifugation (*n* = 4). (**C**) Protein co-precipitation levels quantified by bicinchoninic acid assay (BCA) assay showed a low co-precipitated protein in EVs isolated by ExoGAG compared to those isolated by ultracentrifugation (** *p* = 0.0011 according to paired *t*-test; *n* = 4). (**D**) Representative transmission electron microscopy (TEM) images of purified EVs harvested by ultracentrifugation (left panels) and ExoGAG (right panels), further coupled to AuNP-PEG-CD9 as shown in the magnifications.

First, we applied the EVs purification technology to separate and characterize the fraction of tumor EVs from conditioned cell culture medium from Hec1A endometrial cancer cells. Hec1A culture media, supplemented with fetal bovine serum (FBS) reduced in EVs, was recovered upon conditioning for 48 h and processed with ExoGAG following the manufacturer’s instructions. Briefly, the recovered conditioned media was incubated for 5 min with the ExoGAG reactive and centrifuged at 3000× *g* for 30 min. The pellet containing EVs was then resuspended in PBS and directly analyzed by nanoparticle tracking analysis (NTA) Nanosight NS300 as described in Section 4. As shown in Figure 1A, both the representative image of the purified EVs (left panel) and their particle profile (right panel) were consistent with a homogeneous population of particles of approximately 200 nm, similar to the particle profile of EVs purified by ultracentrifugation at 100,000× *g* for 16 h, performed in parallel as a methodological control. Minor differences in particle size and particle subpopulations between EV sample comparison may be related to the limitations of NTA sensibility for the profiling of biological vesicles [18]. In addition, the efficiency of EVs purification by ExoGAG, expressed as the number of EVs per frame in the precipitate, was similar compared to the number of EVs in the pellet obtained by ultracentrifugation (Figure 1B). By contrast, the quantification of protein contaminants from the ExoGAG methodology of EVs separation by bicinchoninic acid assay (BCA) resulted in an improved purification compared to the ultracentrifugation protocol (** *p* = 0.0011 according to paired *t*-test) (Figure 1C). Consistently, transmission electron microscopy (TEM) imaging showed that samples isolated with ExoGAG contained only EVs and crystallized organic remnants of ExoGAG agent (Figure 1D, right panels), while samples precipitated using ultracentrifugation showed a large amount of unwanted organic residues (Figure 1D, left panels). In terms of morphological characterization, EVs obtained using ExoGAG appeared cleaner and better dispersed compared to those isolated by ultracentrifugation presenting large clustering (Figure 1D). Despite the fact that a crystallized ExoGAG layer on the surface of the EVs was observed (Figure 1D, magnification in right panels), it did not prevent the interaction with AuNPs-PEG-CD9 further characterizing the purified EVs. It is likely that when the sample is dispersed in the medium, ExoGAG remains solubilized but as the sample dries for microscopy visualization it begins to crystallize on the EVs’ surface. Similar results in terms of NTA Nanosight NS300 profile, purification, efficiency, and characterization were obtained when EVs were purified with ExoGAG from the MDA-MB-231 breast cancer cell line, the endometrial cancer cell line Ishikawa, and the ovarian cancer cell line SKOV3 (Figure S1). From these results, we conclude that ExoGAG technology meets the appropriate criteria to obtain a highly purified population of EVs with an optimal particle profile and recovery rate, and a rapid and simple process, thus making it suitable to translate an EV-based biomarker into the clinical setting.

### 2.2. Purification of EVs from Plasma Samples

**Figure 2:**
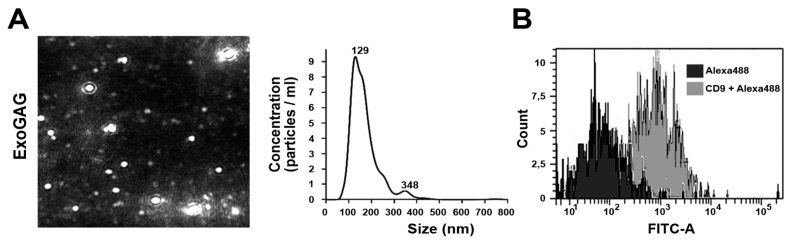
Characterization of plasma EVs isolated by ExoGAG. (**A**) NTA Nanosight NS300 particle tracking profile of harvested EVs expressed in size (nm) and concentration (particles/mL) showed a population of 200 nm in average (*n* = 4). (**B**) Cytometry analysis of EVs resulted in a specific CD9 labeling of the EVs purified (*n* = 2). FITCA: Fluorescein isothiocyanate A.

In order to provide a useful tool to translate EV-based biomarkers into clinics, we next assessed the performance of ExoGAG technology in plasma, as the most suitable sample for liquid biopsy in EC. Plasma samples were processed with ExoGAG as indicated by the manufacturer. Briefly, 500 µL of plasma were incubated with 1 mL of ExoGAG reactive for 5 min before centrifugation at 16,000× *g* for 15 min, resulting in a precipitate containing EVs. The representative NTA Nanosight NS300 image and particle profile of EVs population purified by ExoGAG from plasma showed a homogeneous population of 200 nm on average (Figure 2A). In this case, the concentration of EVs isolated by ExoGAG was higher compared to ultracentrifugation (Figure S2A, left panel). In addition, cytometry analysis resulted in the specific CD9 labeling of EVs purified by ExoGAG (Figure 2B), also similar to those purified by ultracentrifugation (Figure S2A, right panel). We further compared ExoGAG technology with ExoQuick and ExoSpin, two commercially available technologies for the isolation of EVs also based on precipitation agents. As shown in Figure S2B, ExoGAG showed no significant reduction on EV purification efficiency, but significantly reduced levels of co-precipitated contaminants. All these results confirmed the performance of ExoGAG to separate the population of EVs also from a complex plasma sample, with optimal quality, purity, and recovery rate.

### 2.3. ANXA2 Levels Are Increased in Circulating EVs of EC Patients

**Figure 3:**
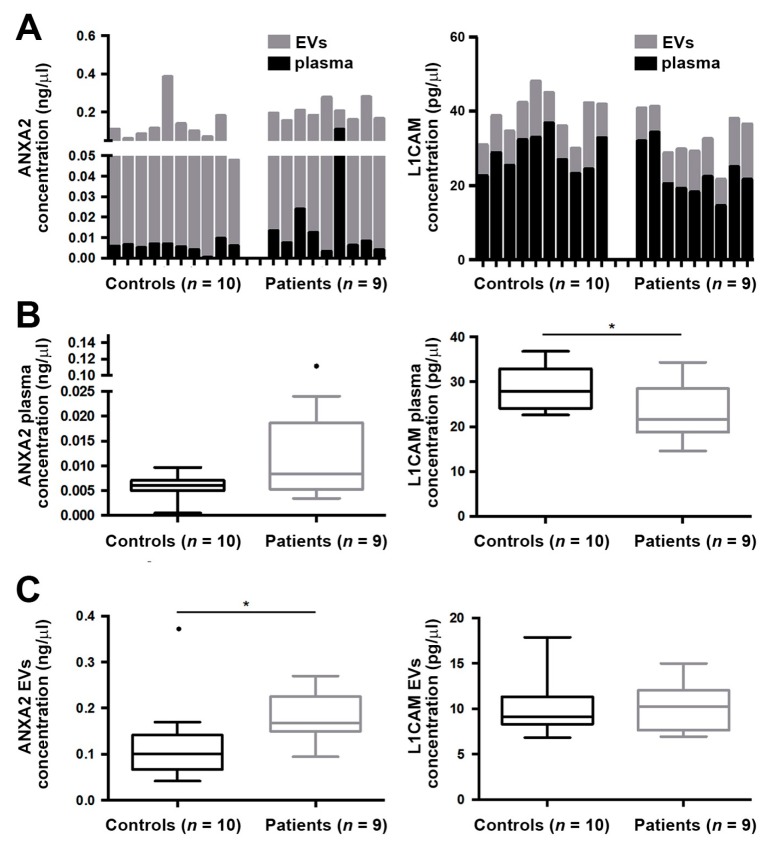
ANXA2 and L1CAM levels in endometrial cancer (EC) plasma samples and circulating EVs from patients and healthy controls. (**A**) Analysis of ANXA2 and L1CAM levels in plasma showed that ANXA2 levels mainly corresponded to purified EVs while L1CAM levels were mainly present in the soluble fraction of plasma. (**B**) Analysis of both markers in complete plasma samples from healthy controls and patients showed higher levels of L1CAM in controls and no significant differences for ANXA2 levels (* *p* = 0.027 and *p* = 0.090, respectively, according to Mann–Whitney U test). (**C**) Analysis of ANXA2 levels in circulating EVs showed significantly increased levels in patients vs. healthy controls (* *p* = 0.027, according to Mann–Whitney U test) while L1CAM did not show differences (*p* = 0.887, according to Mann–Whitney U test). These comparative analyses were conducted with 9 patients and 10 healthy controls included in the global cohort of patients and healthy controls (see Table 1).

We then applied the ExoGAG technology to a cohort of plasma samples from EC patients and healthy controls to analyze its clinical value. Preliminary data indicated that the number of EVs in patients was increased compared to healthy controls (* *p* = 0.0148 according to Mann–Whitney U test) (Figure S3), in accordance with previous data [19]. We then analyzed the protein levels of L1CAM and ANXA2 by ELISA in the purified EVs, known biomarkers with prognostic value in endometrial tumor tissue samples. We first assessed the distribution of the levels of ANXA2 and L1CAM in complete plasma before ExoGAG purification and in the purified EVs after ExoGAG purification in healthy controls (*n* = 10) and patients (*n* = 9). We observed that ANXA2 expression mainly corresponded to the purified EVs, with a residual component of the free soluble protein in plasma (Figure 3A, left panel). By contrast, the distribution of L1CAM levels indicated that the majority of L1CAM content did not correspond to the purified EVs and resulted from the soluble fraction of plasma samples (Figure 3A, right panel). When analyzed for its diagnostic value in this cohort of 9 patients and 10 controls, the levels of ANXA2 biomarker were found increased in complete plasma samples from patients compared to healthy controls, although no statistical significance was observed (*p* = 0.090, according to Mann–Whitney U test); meanwhile L1CAM showed significantly higher levels in healthy individuals (* *p* = 0.027, according to Mann–Whitney U test) (Figure 3B). Interestingly, when we focused the analysis on ExoGAG EVs, significantly higher levels of ANXA2 were found in patients in comparison with the controls (* *p* = 0.027, according to Mann–Whitney U test), while L1CAM did not show differences between groups (*p* = 0.887, according to Mann–Whitney U test) (Figure 3C). These results evidenced the potential of ANXA2 monitoring in EVs isolated from plasma of EC patients using ExoGAG technology.

### 2.4. ANXA2 Levels in Purified EVs from EC Patients Showed Diagnosis Value and Correlated with Tumor Aggressiveness

**Figure 4:**
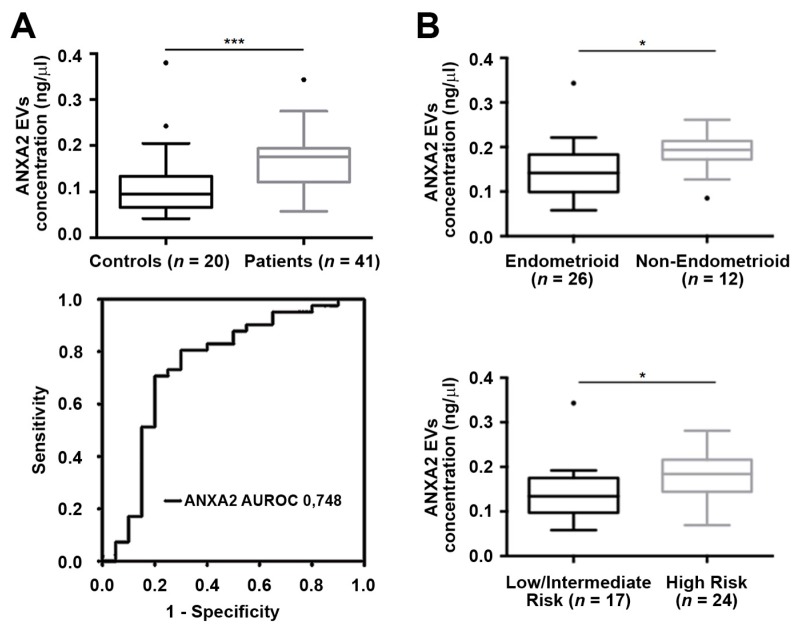
ANXA2 levels in EVs purified by ExoGAG as a diagnosis and prognosis marker in EC. (**A**) Analysis of ANXA2 levels in circulating EVs showed significantly increased levels in patients vs. healthy controls (*** *p* = 0.001, according to Mann–Whitney U test) (upper panel). Receiver operating characteristic (ROC) curves confirmed the power of this biomarker to discriminate EC patients and healthy controls (lower panel). (**B**) ANXA2 levels according to the histology and the risk of recurrence of the EC cohort. Patients with non-endometrioid (*n* = 12) and high risk of recurrence tumors (*n* = 24) showed higher levels of ANXA2 (* *p* = 0.019 and * *p* = 0.012, respectively, according to Mann–Whitney U test). All analyses were conducted on *n* = 41 patients and *n* = 20 healthy controls.

Taking into account the results obtained addressing the EV-associated ANXA2 and L1CAM levels, we decided to further study the value of ANXA2 as a diagnostic marker. For that, we isolated the ExoGAG EVs in a cohort of 41 patients with EC and 20 healthy controls. After this analysis, the increased levels of ANXA2 in the cohort of patients was validated (*** *p* = 0.001 according to Mann–Whitney U test) (Figure 4A, upper panel). We also assessed the clinical value of this marker by performing a receiver operating characteristic (ROC) curve analysis to determine its diagnosis accuracy, obtaining an area under receiver operating curve (AUROC) of 0.74 (*** *p* = 0.002) (Figure 4A, lower panel), which evidenced its accuracy and sensitivity to discriminate the patients’ population. We continued to question whether plasmatic EV-associated ANXA2 levels were linked with a more aggressive disease, as already described for primary tumors. For this, we analyzed the correlation of ANXA2 in EVs purified by ExoGAG from plasma samples with the clinical characteristics of our complete cohort of EC patients. A positive correlation was found between the levels of ANXA2 and the risk of recurrence and the tumor histology, showing those patients with non-endometrioid tumors and high risk of recurrence had higher ANXA2 levels in purified EVs (* *p* = 0.019 and * *p* = 0.012, respectively according to Mann–Whitney U test) (Table 2, Figure 4B). In addition, higher levels of ANXA2 in EVs purified from plasma of Grade 3 and FIGO (International Federation of Gynecology and Obstetrics) stage III–IV EC patients were found (* *p* = 0.038 and * *p* = 0.043, respectively according to Mann–Whitney U test) (Table 2). These results evidenced the potential of EV-based ANXA2 expression as a prognosis liquid biopsy biomarker in EC.

## 3. Discussion

The deficit in appropriate technologies for efficient purification of EVs from complex plasma samples remains the main impediment for the implementation of liquid biopsy-based biomarkers with high specificity and sensitivity in clinical practice [20]. To overcome this limitation, in this work we have combined an efficient EVs purification technology with known biomarkers with clinical value. The ExoGAG technology demonstrates optimal abilities to separate tumor EVs from conditioned culture media of representative EC cells and from the more complex plasma samples from EC patients. This includes adequate particle profile and recovery rates, high purity EVs with minimal protein contaminants, and an easily implementable processing into clinical routine. All these advantages, compared to ultracentrifugation as the gold standard technology, as well as to other commercial precipitation strategies, make this technology adequate for the requirements of specificity and sensibility in EVs purification. Of note, regarding the other precipitation-based strategies analyzed in our study, the main advantage of ExoGAG technology is the purity of the EVs obtained, which is a key factor for their posterior characterization. Linked to the extensive literature showing that EVs derived from tumor cell lines contribute to tumor invasion, chemoresistance, angiogenesis, tumor innervation, metastasis or immune escape [21,22], and to those evidences demonstrating that tumor-derived EVs are associated with advanced disease in different indications [23,24,25,26,27,28] and the response to therapy [29], the analysis of EV-based biomarkers should be expedited for the translation of this form of liquid biopsy into the clinics.

We aimed to validate this relevant technological strategy by selecting L1CAM and ANXA2 as known independent biomarkers with prognostic and predictive value in EC [3,4,6]. The L1CAM cell adhesion molecule is an axonal glycoprotein belonging to the immunoglobulin supergene family. The ectodomain, consisting of several immunoglobulin-like domains and fibronectin-like repeats (type III), is linked via a single transmembrane sequence to a conserved cytoplasmic domain. Importantly, several studies have described the association between L1CAM expression and a poor evolution of EC patients [5,30]. In particular, in early-stage EC with endometrioid histology, expression of L1CAM was related to unfavorable pathological findings, distant recurrence, and poor survival, while in high-grade EC and non-endometrioid EC, L1CAM expression was correlated to an advanced stage and lymphovascular space invasion and distant recurrence [3,31,32]. In this work, we observed that L1CAM expression in plasma samples was mainly present as free soluble and not associated with EVs, resulting in no differences between EC patients and healthy controls.

On the other hand, ANXA2 is a member of the annexin family, a calcium-dependent phospholipid-binding protein family which plays an important role in the regulation of cellular growth and in signal transduction pathways. Furthermore, this membrane protein has been involved in endocytosis and exocytosis processes [33]. More specifically, ANXA2 expression in tissue samples has been associated with a higher risk of recurrence in EC [6]. Importantly, our study evidenced EVs specific expression of ANXA2 in plasma samples from EC patients, with almost no levels of protein expressed as a free soluble protein, demonstrating that EV-based biomarker strategy can improve specificity and sensibility of this biomarker in the prediction of EC outcome. To this regard, the specific expression of ANXA2 has been described to play a role in angiogenesis and metastasis in breast cancer, with promise as a potential biomarker as well as a therapeutic target [34]. Moreover, our results also demonstrate that the levels of ANXA2 in purified EVs from the plasma of EC patients correlate with the tumor histology, grade, stage, and risk of recurrence, reinforcing their interest as a marker linked to more aggressive tumors. This translates the clinical utility of ANXA2 from tissue samples to the liquid biopsy frame and guarantees further studies for early detection of advanced endometrial cancer patients resulting in recurrent disease and for monitoring the response to therapy and early assessment of progressive disease.

## 4. Materials and Methods

### 4.1. Patients

The present study was approved by the Autonomic Galician Ethical Committee (code 2017/430). Patients participating in the study were recruited between January 2018–April 2019 at the Gynaecologic Department of Vall d’Hebron University Hospital (Barcelona, Spain), the MDA Anderson Cancer Centre of Madrid, and the University Hospital of Santiago de Compostela (Santiago de Compostela, Spain). Our EC cohort included low- to high-risk, grade 1–3, and stage I–IV, and recently diagnosed carcinomas or recurrences (Table 1). The control group included a set of 20 healthy women with the absence of a previous cancer episode and with an age range similar to patients (mean: 65.5; range: 47–77 years). In all cases, peripheral blood samples were collected at the time of surgery of the primary tumor. Importantly, the study was carried out following the rules of the Declaration of Helsinki of 1975, revised in 2013. Informed consent approved by the pertinent ethical committees was signed by all patients (Autonomic Galician Ethical Committee code 2017/430).

### 4.2. EV Production and Isolation from Conditioned Medium

The human endometrial cancer cell lines Hec1A and Ishikawa, the ovarian cancer cell line SKOV3, and the breast cancer cell line MDA-MB-231, as representative gynecological aggressive cell lines, were used to test the efficacy of ExoGAG technology. Cancer cell lines Hec1A and SKOV3 were cultured in McCoy’s 5A media (Gibco, Grand Island, NY, USA) and Ishikawa and MDA-MB-231 were cultured in Dulbecco’s Modified Eagle Medium (DMEM) (Lonza, Walkersville, MD, USA), supplemented with 1% penicillin-streptomycin (Gibco, Grand Island, NY, USA) and 10% FBS (Gibco, Thermo Fisher Scientific) reduced in EVs by ultracentrifugation during 16 h at 4 °C, as described in [14] (see Figure S4). Cells were maintained in a humidified atmosphere at 37 °C and 5% CO_2_. After 48 h, the culture medium was recovered and small EVs were harvested by differential centrifugation or ExoGAG.

#### 4.2.1. Differential Centrifugation

Conditioned medium (40 mL) was initially cleared of cell debris (10,000× *g*, 20 min, 4 °C) in a SW32Ti rotor (Beckman Coulter, Brea, CA, USA). EVs were then collected at high-speed ultracentrifugation (100,000× *g*, 16 h, 4 °C) using the same rotor and resuspended in PBS.

#### 4.2.2. ExoGAG

Conditioned medium (40 mL) was incubated with 20 mL of ExoGAG reagent (Nasasbiotech, A Coruña, Spain) for 5 min. EVs were collected by centrifugation (3000× *g*, 30 min, 4 °C; Sorvall ST 8R centrifuge, Thermo Scientific, Osterode am Harz, Germany) and resuspended in PBS.

### 4.3. Isolation of EVs from Plasma

Peripheral blood from EC patients was collected in CellSave tubes (Menarini, Silicon Biosystem, Huntingdon Valley, PA, USA). Plasma was then extracted after a two-step centrifugation at 1600× *g* and 6000× *g* for 10 min. After the second centrifugation plasma was stored at −80 °C until use. EVs were isolated from 500 µL of human plasma samples by ExoGAG. In this case, plasma samples were incubated with ExoGAG reagent (1 mL) for 5 min. EVs were collected by centrifugation at 16,000× *g*, 15 min, 4 °C (Eppendorf, Hamburg, Germany) and resuspended in PBS. Alternatively, EVs were isolated by differential ultracentrifugation as described above (Figure S2A), and with ExoQuick (System Biosciences, Mountain View, CA, USA) and ExoSpin (Cell Guidance Systems, Cambridge, UK) technologies, also based on precipitation agents, following the manufacturer’s instructions (see Figure S2B).

### 4.4. Characterization of EVs

Size and concentration EVs assessed by nanoparticle tracking analysis (NTA) Nanosight NS300 (Malvern, UK) and isolation efficiency was measured in particles/frame. Total EV protein was extracted using lysis buffer (150 mM NaCl, 50 mM Tris-HCl (pH 8.5), 5 mM Ethylenediaminetetraacetic acid (EDTA), 10% Triton ×100, 2 mM Na_3_OV_4_, 4 mM NaF, 1× Phenylmethylsulfonyl fluoride (PMSF), 1× Proteasa Inhibitor Cocktail (PIC)) and protein extracts were quantified by bicinchoninic acid assay (BCA assay).

In flow cytometry analysis (BD FACSAria^TM^ IIu, BD biosciences, San José, CA, USA) we analyzed CD9, CD63, and CD81 protein levels as the most abundant membrane proteins on EVs and usually employed as markers for their characterization [35,36]. EVs isolated from MDA-MB-231 cells were incubated for 1 h with CD81 (1:50, sc7637, Santa Cruz Biotechnology, Santa Cruz, CA, USA) and CD63 (1:50, sc5275, Santa Cruz Biotechnology, Santa Cruz, CA, USA), specifically expressed in this breast cancer cell line [37], followed by anti-mouse Alexa488 (1:1000, ab150113, Abcam, Cambridge, UK) as the secondary antibody. This last antibody was used as a negative control for background fluorescence. EVs purified from plasma were incubated with anti-mouse CD9 antibody (1:50, sc13118, Santa Cruz Biotechnology, Santa Cruz, CA, USA)

EVs were visualized by transmission electron microscopy (TEM) (FEI TECNAI T20, Thermo Fisher Scientific, Waltham, MA, USA). For this purpose, anti-mouse CD9 antibody (sc13118, Santa Cruz Biotechnology, Santa Cruz, CA, USA) was functionalized with gold nanoparticles. Gold (III) chloride hydrate (50% Au basis), sodium citrate tribasic dihydrate (99%), O-(3-Carboxypropyl)-O′-(2-(3-mercaptopropionylamino) ethyl)-polyethylene glycol (Mw. 5000 Da) (SH-PEG-COOH), N-(3-Dimethylaminopropyl)-N′-ethylcarbodiimide hydrochloride (98%) (EDC), N-Hydroxysuccinimide (97%) (NHS), and phosphotungstic acid hydrate were purchased from Merck KGaA (Darmstadt, Germany) and used as received. Citrate-capped gold nanoparticles (AuNPs) were synthesized following a slightly modified version of the Turkevich–Frens method [38] as reported by Lafuente et al. [39]. Afterward, the AuNPs were functionalized with the anti-mouse CD9 antibody via carbodiimide/hydroxysuccinimide chemistry [40] using a heterobifunctional linker. In detail, 1 mL of O-(3-Carboxypropyl)-O′-(2-(3-mercaptopropionylamino) ethyl)-polyethylene glycol aqueous solution (2 mg/mL) was added dropwise into a vial containing 1 mL of the initially prepared AuNP aqueous dispersion (2 mg/mL) and left to react for 12 h under stirring. Then SH-PEG-COOH functionalized AuNPs were washed three times in Milli-Q water and three times in borate buffered saline (pH 9) (BBS). Subsequently, 250 µL of AuNPs-PEG-COOH dispersion in BBS (0.2 mg/mL) were mixed with 25 µL of an antibody solution in BBS (0.2 mg/mL) for 1 h under stirring. Then, 96 µg of EDC and 115 µg of NHS were added to the mixture and let to react for 24 h. Obtained AuNPs-PEG-CD9 were washed three times in tris-borate-EDTA quenching solution reacting for 12 h to block unreacted groups. Finally, the resulting NP was washed three times in phosphate buffered saline (pH 7.4) and stored at 4 °C for further use.

AuNPs-PEG-CD9 were incubated with EVs in PBS for 24 h at 37 °C. Then, samples were separated by centrifugation at 3000 rpm for 5 min and at 13,000 rpm for 15 min. First washing at 3000 rpm was performed to remove large aggregates that could interfere with TEM visualization. A second centrifugation at 13,000 rpm was performed to separate free EVs from free AuNP-PEG-Ab and those that interacted with EVs. All samples were contrasted with a phosphotungstic acid aqueous solution (3 wt. %) and analyzed by imaging using a TEM operated at 200 kV.

### 4.5. ANXA2 and L1CAM Levels in Plasma Samples and Circulating EVs

ANXA2 and L1CAM levels in plasma and EVs were determined by ELISA. For that, a total volume of 500 µL of plasma was used to isolated EVs by ExoGAG as previously described. Total EVs protein was extracted using lysis buffer. ANXA2 and L1CAM protein levels in total plasma and EVs were determined by ELISA (Cloud-Clone Corp, Katy, TX, USA) according to the manufacturer’s instructions. The absorbance was read at 450 nm using a plate reader (Epoch 2 microplate reader, BioTek, Winooski, VT, USA) to quantify the protein content of each marker.

### 4.6. Statistical Analysis

Statistical analyses were conducted using SPSS (version 19.00 for Windows, Chicago, IL, USA) and GraphPad Prism 6.00 software (GraphPad Software Inc., San Diego, CA, USA). EV yield between conditions was analyzed by paired *t*-test. Comparisons between controls and patients and correlations between clinical data and ANXA2 and L1CAM levels were analyzed by Mann–Whitney U test. Outliers were established based on Tukey’s method. The diagnostic power of ANXA2 levels was determined by ROC curve statistics. For all the analysis a *p*-value <0.05 was considered statistically significant.

## 5. Conclusions

The results from this study demonstrated that ExoGAG is a robust, easy to use, and highly efficient technology for the isolation of EVs from different biological fluids such as conditioned culture medium and plasma. Furthermore, ExoGAG technology demonstrated an optimal performance for the clinical implementation of EVs purified from plasma of EC patients. The specific expression of the EC biomarkers ANXA2 in the isolated EVs improved their specificity and sensibility as liquid biopsy. Moreover, we could observe high ANXA2 levels in plasmatic EVs associated with non-endometrioid tumors and high risk of recurrence tumors, evidencing the potential use of EV-based ANXA2 expression as a diagnostic and prognostic liquid biopsy biomarker in EC.

## Figures and Tables

**Table 1:** Clinical characteristics of the EC cohort.

Feature	*n* (%)	Feature	*n* (%)
**Age (mean, range)**	68 (46–88)	**Myometrial infiltration**	
**FIGO stage**		<50%	17 (41.5%)
I	26 (63.4%)	≥50%	23 (56.1%)
II	6 (14.6%)	Unknown	1 (2.4%)
III	8 (19.5%)	**Risk of recurrence**	
IV	1 (2.5%)	Low	11 (26.8%)
**Histologic type**		Intermediate	6 (14.6%)
Endometrioid	26 (63.4%)	High	24 (58.7%)
Non-endometrioid	12 (29.3%)		
Other	3 (7.3%)		
**Histological grade**			
Grade 1	15 (36.6%)		
Grade 2	11 (26.8%)		
Grade 3	14 (34.1%)		
Unknown	1 (2.5%)		

FIGO, Federation of Gynecology and Obstetrics.

**Table 2:** Circulating EV-associated ANXA2 levels according to the clinical characteristics of EC patients.

Fiture	*n*	ANXA2 (ng/uL) Mean (SD)	*p*
**Age**			
<68	19	0.163 (0.059)	
≥68	22	0.179 (0.066)	0.23
**FIGO**			
I/II	32	0.157 (0.059)	
III/IV	9	0.199 (0.058)	**0.043**
**Histologic type**			
Endometrioid	26	0.150 (0.061)	
Non-endmetrioid	12	0.189 (0.051)	**0.019**
**Histologic grade**			
Grade 1–2	26	0.168 (0.063)	
Grade 3	14	0.191 (0.054)	**0.038**
**Myometrial infiltration**			
<50	17	0.158 (0.066)	
≥50	23	0.175 (0.059)	0.385
**Risk of recurrence**			
Low/intermediate	17	0.147 (0.070)	
high	24	0.188 (0.049)	**0.012**

SD, standard deviation; *p* was calculated according to Mann–Whitney U test. *p* < 0.05 are marked in bold.

## Supplementary Materials

The following are available online at https://www.mdpi.com/2072-6694/11/12/2000/s1, Figure S1: Characterization of MDA-MB-231, Ishikawa, and SKOV3 EVs isolated by ExoGAG; Figure S2: Characterization of plasma EVs isolated by ultracentrifugation, ExoQuick, and ExoSpin; Figure S3: Efficiency (particles/frame) of EVs purified by ExoGAG in a representative cohort of endometrial cancer patients (*n* = 8) and healthy controls (*n* = 8); Figure S4: Efficiency of ultracentrifugation (16 h) to reduce the EVs contain from culture media analyzed by NTA Nanosight NS300 and expressed as particles/frame.
