# Supplementary material for: Extracellular Vesicles-Based Biomarkers Represent a Promising Liquid Biopsy in Endometrial Cancer
Source: Cancers (Basel). 2019 Dec 12;11(12):2000. doi: 10.3390/cancers11122000 (PMC6966595; doi:10.3390/cancers11122000)
Supplement: Supplementary file 1 [file cancers-11-02000-s001.pdf]

cancers

MDPI

Extracellular Vesicles-Based Biomarkers Represent a Promising Liquid Biopsy in Endometrial Cancer

Carolina Herrero, Alexandre de la Fuente, Carlos Casas-Arozamena, Victor Sebastian, Martin Prieto, Manuel Arruebo, Alicia Abalo, Eva Colás, Gema Moreno-Bueno, Antonio Gil-Moreno, Ana Vilar, Juan Cueva, Miguel Abal and Laura Muinelo-Romay

Supplementary Figures

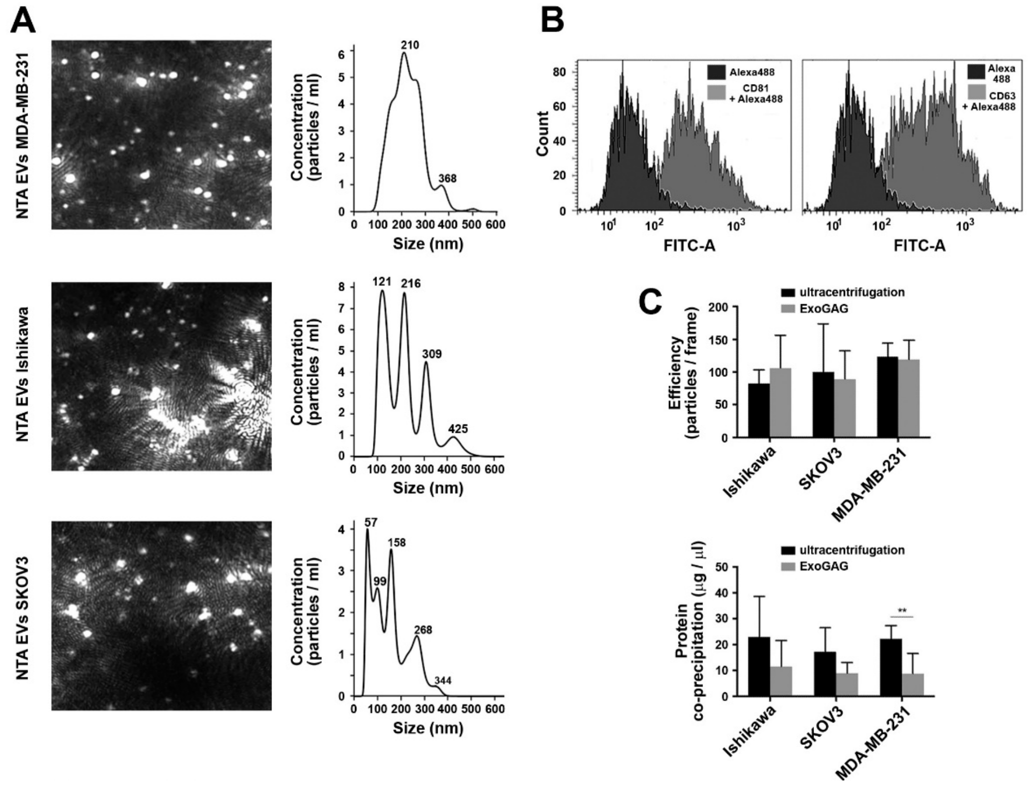

Figure S1. Characterization of MDA-MB-231, Ishikawa and SKOV3 EVs isolated by ExoGAG. **(A)** Representative NTA Nanosight NS300 image of isolated MDA-MB-231 EVs (upper panel), Ishikawa EVs (middle panel) and SKOV3 EVs (lower panel) expressed in size (nm) and concentration (particles/ml) (n = 3); **(B)** Cytometry analysis of MDA-MB-231 EVs resulted in a specific CD81 and CD63 labelling (n = 2); **(C)** Efficiency of purified EVs, expressed as EVs particles per frame, was found similar in ExoGAG and ultracentrifugation in all three cell lines (upper graph) (n = 3). Protein co-precipitation levels quantified by BCA assay showed a low protein co-precipitation in EVs isolated by ExoGAG compared to those isolated by ultracentrifugation in Ishikawa, SKOV3 and MDA-MB-231 EVs ($p = 0.081$, $p = 0.064$, ** $p = 0.005$, respectively, according to paired T-test; n = 4) (lower graph).

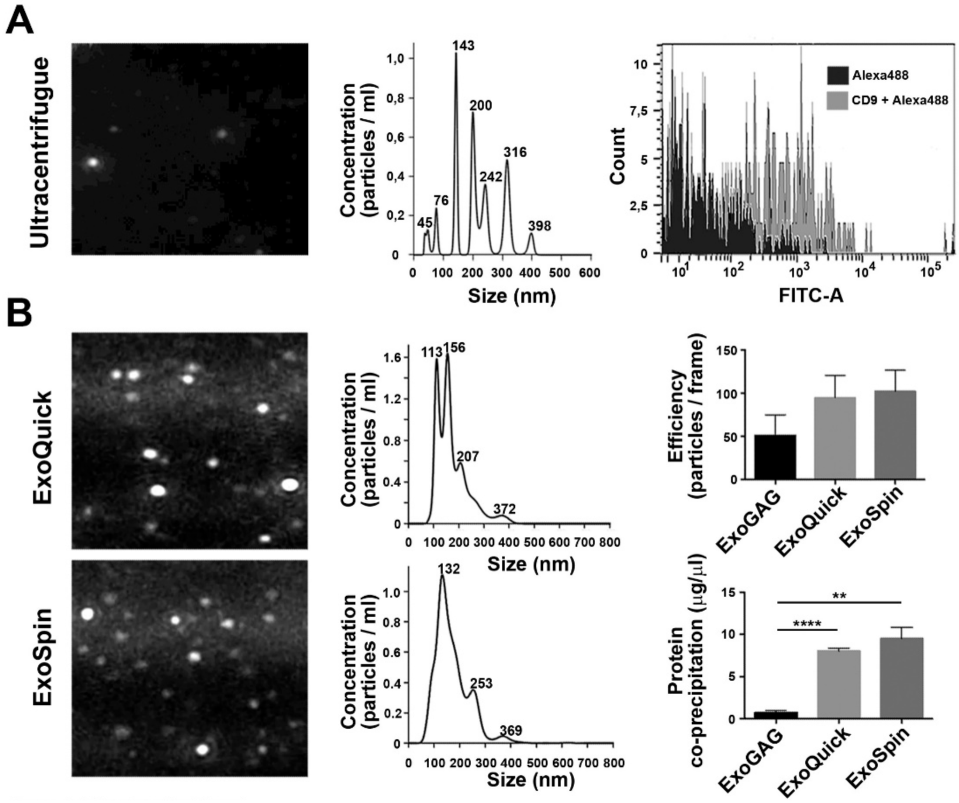

Figure S2. Characterization of plasma EVs isolated by ultracentrifugation, ExoQuick and ExoSpin. **(A)** Ultracentrifugation: EVs were collected at high speed ultracentrifugation (100,000 × g, 16 h, 4 °C) in a SW32Ti rotor (Beckman Coulter, Brea, CA, USA) and resuspended in PBS. NTA Nanosight NS300 particle tracking profile of plasma EVs isolated by ultracentrifugation expressed in size (nm) and concentration (particles/ml) resulted in a low efficiency of recovery (left panel); n = 2. Cytometry analysis showed a specific CD9 labelling of EVs purified (right panel) (n = 2); **(B)** ExoQuick: 50 µL of plasma samples were incubated with 13.4 µL of ExoQuick reagent during 30 min and collected by centrifugation at 3000 × g, 10 min, 4 ºC (Eppendorf, Hamburg, Germany). ExoSpin: 50 µL of plasma samples were incubated with 25 µL of ExoSpin reagent for 5min and collected by centrifugation at 16000 × g, 30 min, 4 ºC (Eppendorf, Hamburg, Germany). NTA Nanosight NS300 particle tracking profile of plasma EVs isolated by ExoQuick and ExoSpin expressed in size (nm) and concentration (particles/ml) (n = 3) (left panel). Efficiency of purified EVs, expressed as the number of EVs per frame, was higher although comparable in ExoQuick and ExoSpin referred to ExoGAG (n = 3) meanwhile protein co-precipitation levels quantified by BCA assay showed a low co-precipitation in EVs isolated by ExoGAG compared to those isolated by ExoQuick and ExoSpin (**** $p < 0.0001$ and ** $p < 0.0014$, respectively, according to paired T-test; n = 4) (right panel).

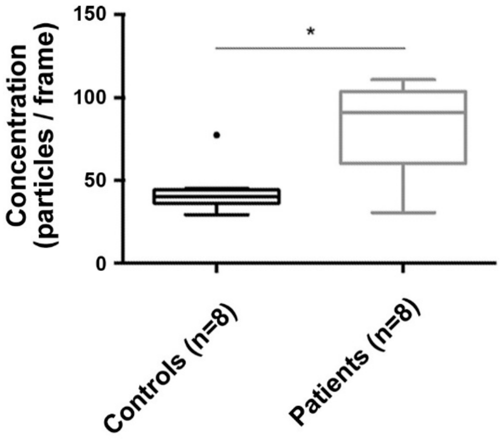

Figure S3. Efficiency (particles/frame) of EVs purified by ExoGAG in a representative cohort of endometrial cancer patients (n = 8) and healthy controls (n = 8). EVs particles levels were significant higher in patients vs. healthy controls (* $p = 0.0148$ according to Mann-Whitney U-test).

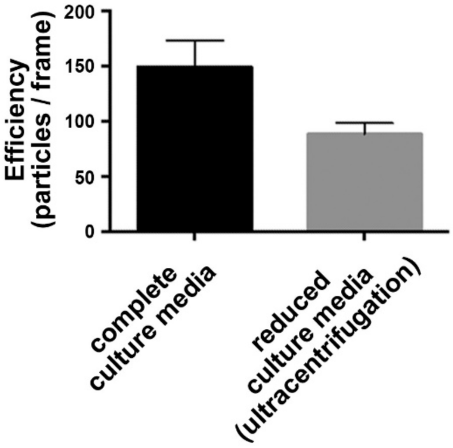

Figure S4. Efficiency of ultracentrifugation (16h) to reduce the EVs contain from culture media analysed by NTA Nanosight NS300 and expressed as particles/frame. Complete culture media resulted in a mean of 149.4 particles/frame whereas reduced culture media resulted in 88.85 particles/frame on average (n = 2).

© 2019 by the authors. Licensee MDPI, Basel, Switzerland. This article is an open access article distributed under the terms and conditions of the Creative Commons Attribution (CC BY) license (http://creativecommons.org/licenses/by/4.0/).
